# Macrophage Related Chronic Inflammation in Non-Healing Wounds

**DOI:** 10.3389/fimmu.2021.681710

**Published:** 2021-06-16

**Authors:** Meirong Li, Qian Hou, Lingzhi Zhong, Yali Zhao, Xiaobing Fu

**Affiliations:** ^1^ Research Center for Tissue Repair and Regeneration Affiliated to the Medical Innovation Research Division and 4^th^ Medical Center, PLA General Hospital and PLA Medical College, Beijing, China; ^2^ PLA Key Laboratory of Tissue Repair and Regenerative Medicine and Beijing Key Research Laboratory of Skin Injury, Repair and Regeneration, PLA General Hospital, Beijing, China; ^3^ Research Unit of Trauma Care, Tissue Repair and Regeneration, Chinese Academy of Medical Sciences, Beijing, China; ^4^ Central Laboratory, Trauma Treatment Center, Central Laboratory, Chinese PLA General Hospital, Hainan Hospital, Sanya, China

**Keywords:** chronic inflammation, chronic wound, macrophage, heterogeneity, aging, diabetes, multidimensional analysis

## Abstract

Persistent hyper-inflammation is a distinguishing pathophysiological characteristic of chronic wounds, and macrophage malfunction is considered as a major contributor thereof. In this review, we describe the origin and heterogeneity of macrophages during wound healing, and compare macrophage function in healing and non-healing wounds. We consider extrinsic and intrinsic factors driving wound macrophage dysregulation, and review systemic and topical therapeutic approaches for the restoration of macrophage response. Multidimensional analysis is highlighted through the integration of various high-throughput technologies, used to assess the diversity and activation states as well as cellular communication of macrophages in healing and non-healing wound. This research fills the gaps in current literature and provides the promising therapeutic interventions for chronic wounds.

## Introduction

Skin wound repair is a critical process for the restoration of skin integrity. It is composed of three sequential and over-lapping wound healing phases: inflammation, proliferation and remodeling (1). An inflammatory phase involves clot formation, platelets released factors to attract neutrophils and macrophages infiltration into the wound and the phagocytic removal of bacteria and debris (2–4). Since then, the wound is cleaned and get ready for tissue regrowth. Precisely programmed initiation and resolution of inflammatory stage is necessary to pursue tissue repair (5, 6). In the proliferative phase, wound cells include keratinocytes, endothelial cells, and fibroblasts, cover and fill the defect through proliferation and migration, and participate in re-epithelization, revascularization and extracellular matrix (ECM) deposition, respectively. In the remodeling phase, the myofibroblasts undergo apoptosis and newly formed granulation tissues are reorganized, and then lead to tissue homeostasis (7–11). However, interruptions or defects in these delicate phases may lead to a non-healing wound state (6, 12).

The prevalence of chronic wounds increases each year and is associated with a variety of conditions, such as old age, obesity, vascular disease, and diabetes (13). There were approximately ∼4.5, 9.7, and 10 million patients suffering from pressure, venous, and diabetic ulcer wound worldwide in recent years. The delay in wound repair has serious effects on patients’ quality of life and poses a significant burden on patient’s caretakers (14). Currently, well-established standard wound care for chronic wounds include pressure off-loading, removal of necrotic tissue, pathogenic suppression, and topical wound dressings (15, 16). And advanced strategies include growth factor-based therapy [platelet-derived growth factor (PDGF)], biological dressings (acellular extracellular matrices and cell-containing skin substitutes) are also available for wounds with poor response to standard care. Although the various treatments for chronic wound, there is still a significant number of patients suffer from lower limb amputation due to the further deterioration of the wounds (17). Even more unfortunately, the occurrence of chronic wounds is rises at a higher rate than the emergence of novel and effective treatment strategies.

Chronic wounds are defined as wounds stalled in a constant and excessive inflammatory state (10). Considerable evidence revealed that chronic wounds are closely associated with impaired phenotype transition of pro-inflammatory macrophages to anti-inflammatory phenotypes in wounds (10, 18–20). Macrophages play essential roles in the orchestration of transitions among three healing phases. Their phenotype readily changes according to spatiotemporal cues during repair (21). Many functional attributes ascribed to macrophages are found in skin wound models, such as scavenging, phagocytosis and antigen presentation in inflammatory phase (22), stem cell recruitment and revascularization in proliferative phase (23), and extracellular signaling transduction in remodeling phase, owing to their plasticity and heterogeneity (24). Recently, particular attention has been paid to their resolution of inflammation and shift toward regeneration (25). And macrophages were also recognized as “background actor” that participate in the immunomodulation in numerous diseases (26). This immune cell population was considered as a promising therapeutic target and can potentially be manipulated in a tissue-specific manner. For example, pro-inflammatory macrophage depletion have a universal protective effect on acute kidney injury (27). Monoclonal antibodies to inhibit macrophagic pro-inflammatory pathways achieve therapeutic efficacy in osteoarthritis (28). Therefore, a review of the plasticity, flexibility, and heterogeneity of macrophages under physiological and pathological conditions can not only provide clues to enable modification of improper kinetics in the macrophage response in chronic wounds, but also contribute to the understanding and identification of novel therapeutic targets for other injured tissues.

In this review, we first present the origin, function, and heterogeneity of wound macrophages. Subsequently, we discuss factors that contribute to the impaired activation and functions of macrophages in non-healing wounds. We highlight current biotherapy methods for restoring the function of macrophages. Finally, important molecular targets and novel macrophage-based treatment for chronic wounds are proposed from the perspective of single-cell sequencing.

## Macrophage Biology in Wound Healing

Macrophages in wounds originate from two primary sources—tissue resident and bone marrow, the latter occupy a larger proportion and play dominant roles in wound repair (29–31). Their essential roles in wound healing have long been well-established in classical macrophage depletion model. A transgenic mouse model of inducible macrophage depletion revealed a prolonged inflammation, disturbed neo-vascularization, impaired fibroblast differentiation and delayed healing after non-selectively abrogate macrophages in wounds, which supported the notion that macrophages were key regulators to ensure proper healing (32, 33). And the selective depletion of macrophage at different stages of wound repair implied that macrophages took on distinct roles throughout the healing process (34).

### Roles of Macrophages in Inflammation Phase

Resident and recruited macrophages play essential roles in the tissue injury response (35). After an injury, resident dermal macrophage are the earliest responders acting to induce the inflammatory response through the release of hydrogen peroxide, which leads to sequentially recruit blood neutrophils and monocytes. Subsequently, recruited monocytes further differentiate into macrophages under NADPH oxidase 1 and 2 (NOX1 and NOX2) (30, 31, 36, 37). During early wound healing, macrophage exhibits an inflammatory phenotype, known as the classically activated M1 macrophage. They act as the first line of defense against pathogen through mainly two ways, namely, recognizing pathogen-associated modifying proteins (PAMPs) on the surfaces of bacteria or fungi to form phagolysosome, and releasing antibacterial mediators, such as reactive oxygen species (ROS) and reactive nitrogen species (38, 39). Moreover, they are involved in removal of cellular debris and clearing apoptotic neutrophils (40). And they produce numerous pro-inflammatory cytokines and chemokines, such as the tumor necrosis factor-α (TNF-α), interleukin-1β (IL-1β), monocyte chemoattractant protein-1 (MCP-1), and chemokine (C-C motif) ligand 2 (Ccl2), to attract defense components and stimulate proliferation of wound cells, such as fibroblasts and keratinocytes. Furthermore, M1 macrophages seem to involve in the initiation of angiogenesis through secretion of angiogenic stimulators including vascular endothelial growth factor (VEGF), basic fibroblast growth factor (FGF2), IL-8, and CCL5 (41).

### Roles of Macrophages in Proliferation Phase

In the following phase, the classically activated M1 phenotype gradually skews toward an alternatively activated M2 phenotype, which is a determining event for the transition from the inflammation phage to the proliferation phase (42–45). The M2 phenotype is known as the healing-associated macrophage with downregulated inflammatory factors and upregulated anti-inflammatory cytokines, including TGF-β, IL-10, and growth factors, such as platelet-derived growth factors (PDGF), resistin-like molecule-α (Relmα), epidermal growth factor, and vascular endothelial growth factor-α (Vegfα), that drive tissue repair (18, 20, 46, 47).

**Figure 1:**
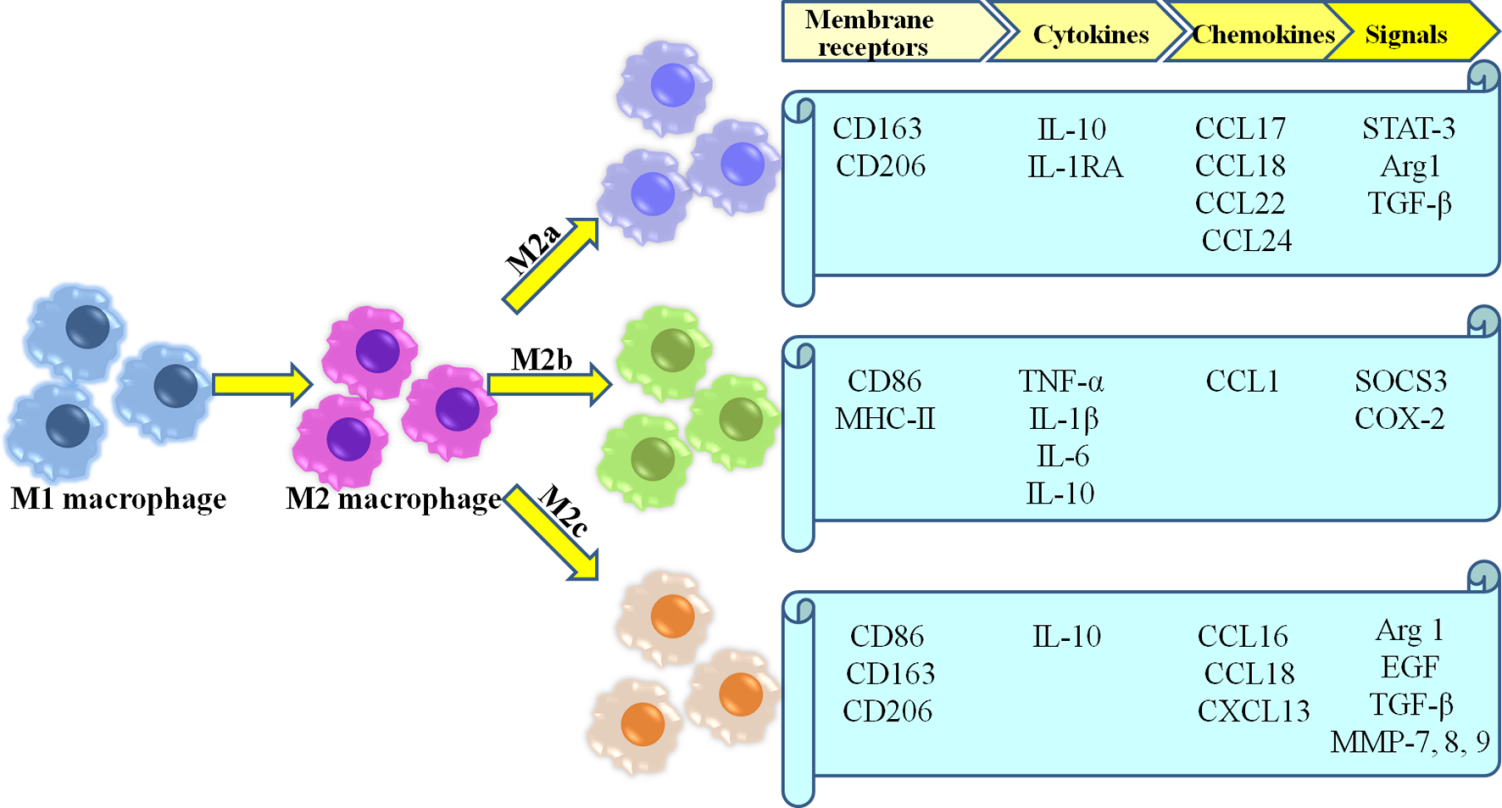
Most common characteristics of M2 macrophages subgroups.

Because of the high plasticity of macrophage, M2 macrophage involved in healing are currently further categorized into three different subtypes (M2a, M2b,and M2c) (35, 46, 48, 49) (**Figure 1**). M2a macrophages become more prevalent as wound healing progresses, and produce high levels of arginase-1 (Arg-1), PDGF, insulin-like growth factor-1 (IGF-1) and other cytokines (50). Moreover, as a pro-fibrotic phenotype, they directly secrete the fibrosis-related proteins contributing to collagen deposition in wounds (51–53). They also serve to promote angiogenesis, as well as proliferation, migration, and differentiation of fibroblasts (54). M2b macrophages are considered as the mixed regulatory phenotype which balance anti-inflammatory and pro-inflammatory functions, due to their production of pro-inflammatory cytokines (e.g., TNF-α, IL-6, and IL-1) as well as large amounts of anti-inflammatory cytokine IL-10 along with low levels of IL-12 (55, 56). Moreover, M2b macrophages derived exosomes are abundant mRNAs encoding proinflammatory cytokines, chemokines, and regulation factors compared with M2a macrophages (57). Thus, M2b macrophages may represent an intermediate between M1 and M2a polarization states (58). M2c macrophages are considered as tissue repair macrophages which exhibit strong anti-inflammatory activities by releasing large amounts of IL-10 and augmenting the behavior of regulatory T-cells (6, 59, 60). Furthermore, they play a role in angiogenesis through increased endothelial cell migration and tube formation (41, 61). Additionally, phagocytosis of wound debris, and the deposition of ECM components has also been demonstrated in M2c macrophages (54).

M2 macrophages also recruit stem cells to wounds, which are an important driving force in tissue repair, exerting tissue reparative effects through paracrine signaling (62, 63). Silva et al. showed that macrophage-derived GPNMB promotes the polarization of the M1 phenotype into the M2 phenotype (64). The later facilitates the mobilization of mesenchymal stem cells (MSCs) to the wound, thereby improving wound healing (65). However, the subpopulation of M2 macrophages that plays a role in the recruitment of MSCs has not been identified to date. Moreover, M2c macrophages have been shown to secrete MMP-9 to attract vessel associated, blood-derived stem cells to fulfill the above-mentioned function of angiogenesis in injured sites (66).

### Roles of Macrophages in Remodeling Phase and Regeneration

Prolonged activation of M2 macrophages can lead to excessive wound healing and ultimately fibrosis (67). Therefore, the number of macrophages starts to decline during the remodeling phage. The remaining macrophage play a role in tissue remodeling (68). Matrix metalloproteinase are major proteolytic enzymes involved in the turnover of the extracellular matrix (ECM) during cutaneous wound repair. MMP-10 is derived from macrophage shaped ECM deposition indirectly by upregulation of MMP-8 and MMP-13 (52). Furthermore, the mannose receptor-mediated endocytic pathway for degradation collagen was revealed as another dominant path for collagen turnover by M2 macrophages (69).

Previous research links macrophages to hair follicle growth, as perifollicular resident macrophages prompt the entry of hair follicle (HF) stem cells into the anagen phase of growth through Wnt-related apoptosis signaling (70, 71). Full-thickness skin wounds usually leaves hairless scars, and macrophages are important for regeneration. The study of Martinot et al. showed that hair-bearing areas heal faster than areas lacking HFs (72). Recent studies showed that M2 macrophages release growth factors, such as the hepatocyte growth factor (HGF) and insulin-like growth factor 1 (IGF-1) to activate stem cells and facilitate HF regeneration in a skin mechanical stretch model (73). Results by Wang et al. revealed that M2 macrophages activate HF stem cells in the later stage of wound healing, leading to a telogen–anagen transition around the wound and *de novo* HF regeneration in wound-induced hair follicle neogenesis (WIHN) (74). Osaka et al. found that the apoptosis signal-regulating kinase 1 (ASK1) is needed for the regulation of infiltration and activation of macrophages, and required for macrophage-dependent hair regrowth in wounding-induced hair regrowth (75). However, the phenotypes of macrophage subgroups associated with HF regeneration must be further confirmed, and the emergence timing and the quantity of this specific subgroup may directly determine whether the HF can regenerate. Furthermore, there is lacking evidence on whether the regeneration of other appendages in the injured sites, such as sweat glands and sebaceous glands, are closely associate with local macrophages.

In summary, macrophages in wounds act as the “Monkey King” in Chinese myths. They change according to their surrounding environment and needs, and perform their due functions. Therefore, selective targeting of macrophage subpopulations for pro-healing therapy may provide an attractive strategy in regenerative processes. However, the understanding of spatiotemporal cues of each subpopulation of macrophages during the repair process is still limited. We must further explore the plasticity of macrophages and discover more functional subgroups to enable better regulation of the repair process to promote wound repair and regeneration.

## Systemic and Local Factors of Macrophage Dysfunction in Non-Healing Wounds

**Figure 2:**
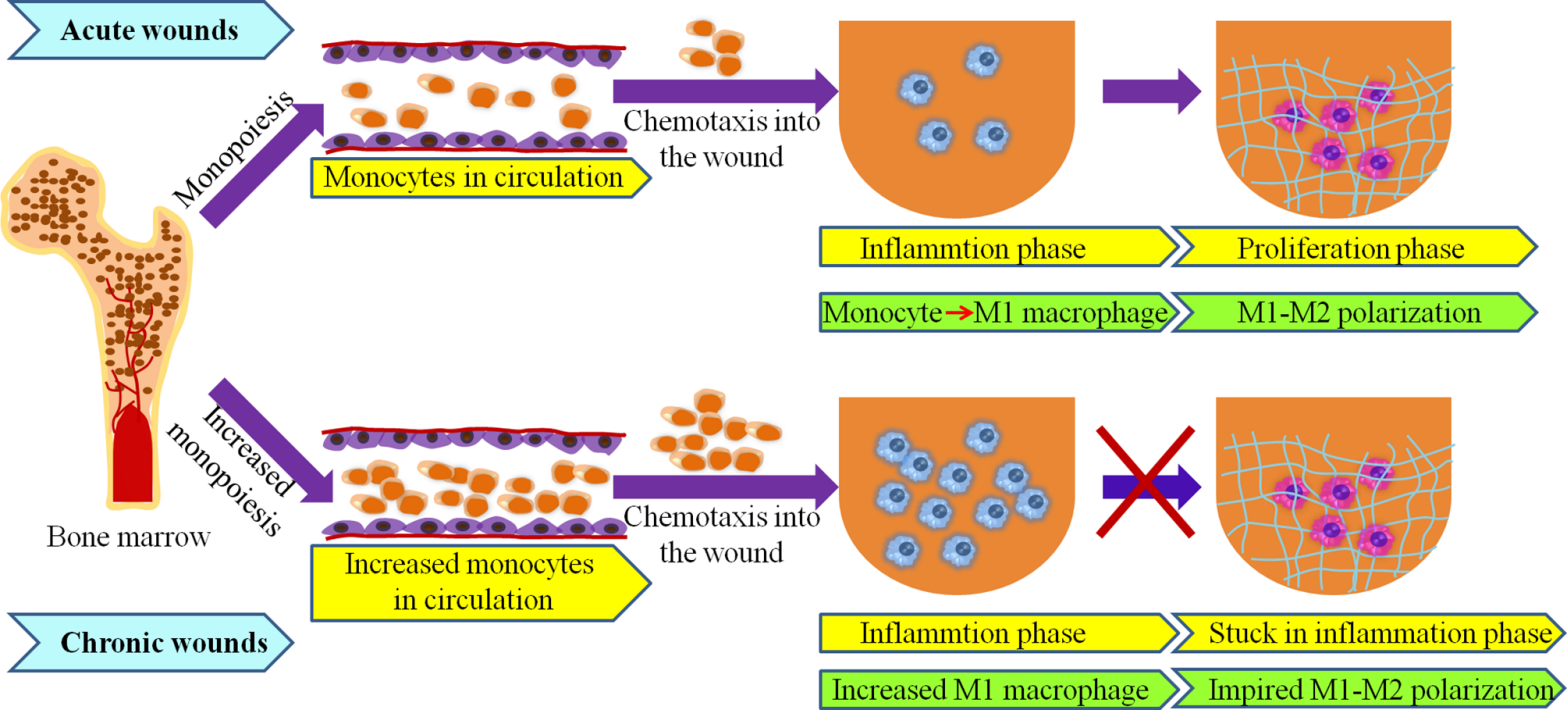
Macrophages originate from bone marrow in acute and chronic wounds. High numbers of bone marrow-produced monocytes under elderly, obese and diabetes may lead to increased number of macrophage in chronic wound. Transition from M1- to M2- phenotypes is impaired in chronic wounds.

As wounds heal, the tight regulation of the macrophage phenotype switching from a M1-proinflammatory to a M2-anti-inflammatory (pro-healing) phenotype contribute to the smooth progress of the repair process (76–78). However, under pathological condition, such as ageing, obesity, infection, and diabetes, M1 macrophages in wounds were restrained with an incomplete switch to M2 phenotype, resulting in the stall of the repair process at inflammatory phase (10, 79, 80) (**Figure 2**). The mechanisms behind the persistent inflammatory macrophage phenotype in chronic wounds have been gradually identified.

### Systemic Factors of Macrophage Dysfunction

Multiple studies reported that macrophages are prone to dysregulation under pathophysiological conditions such as ageing, obesity, and diabetes, and their aberrant activities are present not only at the wound site, but also in bone marrow (BM) and blood circulation (2, 81–83). Alteration in the number and function of macrophage have been revealed in chronic wounds, which are closely related to hematopoietic disruption and particularly to myeloid skewing (67, 84–86). This is known as increased monopoiesis (**Figure 2**).

In aging mice, the hematopoietic stem and progenitor cells (HSPCs) showed restricted diversity and a preferential myeloid-biased differentiation, which was reflected by enhanced accumulation of the myeloid progenitor in aged mice (87). The comparative analysis of expression profiles of HSPCs suggested that the myeloid differentiation related genes in aged mice, such as *Runx1, Hoxb6*, and *Osmr* are upregulated while lymphopoiesis genes are downregulated compared with those of young mice (88). Dramatic changes in the HSPCs milieu or niche in aging mice were attributed to the aging process and myeloid differentiation bias. Niche factors, such as proinflammatory cytokine RANTES, secreted from aging stromal cells and differentiated blood cells have been considered as a major factor in this HSPCs subtype shift (89, 90). Ergen et al.’s study showed that RANTES knockout mice rescued aging-associated myeloid-biased lineage differentiation (89).

Obesity and aging share many common traits in term of immunity and metabolism (91, 92). Several studies showed that obesity modifies the composition of adipose tissue in BM and restrains the generation of osteoblastic cells from mesenchymal progenitors, thereby affecting BM homeostasis. Moreover, adipocytes in BM are key limiting factors of the hematopoietic function in obesity through direct and indirect means (84, 93). Obesity induced oxidative stress is a key driver for the aberrant HSPCs activity skewed toward the granulocyte-macrophage progenitors *via* dysregulation of the expression of Gfi1 in HSPCs (84, 86). Additional studies indicate that an epigenetic-based mechanism is involved in the programming of macrophages biased toward a proinflammatory phenotype under obesity. HSPCs display increased levels of Jmjd3, a member of the JumanjiC (JmjC) family of histone demethylases, which responds to the decreased H3K27me3 at the promoter region of proinflammatory IL-12 (94). This epigenetic feature is passed down to the macrophages at the wound sites, making them predisposed toward a proinflammatory phenotype (94).

Preprogrammed HSPCs committed towards the myeloid lineage and increased proportions of BM myeloid progenitors have also been found in diabetic mice during homeostasis and following injury, which results in an enhanced myeloid output (95–97). An elevated number of circulating monocytes was also revealed in diabetic patients (2). Furthermore, the higher potential to form granulocyte/macrophage colony forming units in BM isolated cells, and the increased number and proliferation of granulocyte-macrophage progenitors and common myeloid progenitors have also been found in diabetes (98). These results suggest a mechanistic link between neutrophils and monocytes. Research conducted by Nagareddy et al. showed that the depletion of neutrophils normalized the monocyte level in diabetic mice. Mechanistically, neutrophils produced more S100A8/A9 which interacted with glucose- inducible receptor for advanced glycation end products (RAGE) on myeloid progenitor cells resulting in the enhanced release of monocytes under hyperglycemia. Moreover, S100A8/A9-initiated monocytosis was dampened when blood glucose drops back to normal (98). Furthermore, hyperglycemia induced higher expression of tyrosine hydroxylase and produced more catecholamines in the spleen leukocyte of diabetic patients and mice, *via* an interaction with the β2 adrenergic receptor on the granulocyte macrophage progenitors, leading to enhanced monocytosis (99). However, the opposite trend was observed in the study by Yan et al. This suggests that T2DM causes reduced differentiation of HSPCs towards monocytes/*macrophages through Dnmt1-dependent repressive* modifications of myeloid lineage associated genes such as Notch1, PU.1 and Klf4 (100).

The above findings demonstrate that the wound M1-dominant macrophage phenotype is set at the BM level. The microenvironment in the BM changes significantly under pathological conditions, such as aging, obesity, and diabetes, which predetermines the gene expression of HSPCs and dysregulates their differentiation potential and function. These aberrant signatures are preserved and passed down to monocytes, which further disrupt the polarization of macrophages and imbalance the M1/M2 phenotype throughout the course of wound healing. Therefore, these findings reveal a novel therapeutic approach for chronic wounds. Therapies target systemic changes in the BM niche and regulate monopoiesis of hemopoietic stem cells (HSCs) to influence peripheral phenotypes and restore the balance of M1/M2 macrophages, which may improve the state of non-healing wounds.

### Local Factors of Macrophage Dysfunction

Presently, there are two theories regarding changes in the number of macrophages in chronic wounds. One is that a large number of macrophages infiltrate in chronic wounds as the results of increased monopoiesis, accompanied by an increase mobilization to the bloodstream. The other is that the number of macrophages is significantly reduced during the inflammatory phase in diabetic mice, which is attributed in part to a weakened chemotaxis into the wound (65, 101). The inconsistency in wound macrophage numbers between studies could result from technical differences in the evaluation of wound macrophage and requires further research. Nevertheless, in either case, chronic wounds are often described as being “stalled” in the inflammatory phase with an impeded M1/M2 phenotype transition at the later stages of wound healing. Several reports showed that this was not only related to the functional modifications in HSCs that are passed down to macrophage progeny, but that it was also associated with local effects mediated by the wound microenvironment (2, 43, 102).

The wound microenvironment has a predominant role on the behavior and functionality of healing cells. Macrophages are highly plastic and can change their phenotype and functions in accordance with local microenvironmental signals (103). According to the non-healing wound research, a large number of studies focused on diabetes-related chronic wounds. Both mouse and human diabetic wound conditioned media prefer to induce a proinflammatory macrophage phenotype of BM- derived macrophage *in vitro*, which suggests an imbalanced microenvironment (43, 44). The main cause of macrophage dysfunction with an increased M1/M2 macrophage ratio is composed of two main factors: (1) dysfunction of macrophages under pathological conditions; (2) numerous microenvironmental biomolecules.

#### Dysfunction of Macrophages Under Pathological Conditions

Cell senescence is a normal physiological process in the repair process. It promotes healing *via* releasing PDGF-AA (104). Conversely, it prevents fibrosis by driving the senescence of myofibroblasts (105). The rapid and effective clearance of these cells is essential for optimal repair outcomes (106, 107). In contrast, an excessive amount of senescent cells in the wound or a disturbance of senescent cell clearance may lead to impaired wound healing.

Similar to those found in wounds of elderly, a large amount of senescent cells accumulate in skin wounds of diabetic mice, and macrophages account for a large proportion of these cells (108). Wilkinson et al. found that macrophages derived from wounds of diabetic animals showed reduced polarization potential and prolonged inflammation, and generally presented a senescent phenotype with secretion of a senescence-associated secretory profile (SASP) (108). SASP is an important approach for a small number of senescent cells in tissues to exert significant local biological effects, and it is implicated in the occurrence of numerous chronic diseases (109–111). For example, proinflammatory SASP is thought to be involved in the development of insulin resistance and type II diabetes mellitus in both mice and humans (112, 113). Wilkinson et al., found that macrophages derived from diabetic murine exhibited senescence phenotype and reduced M2-polarization. And the SASP secreted by macrophages derived from wounds of diabetic mice was enriched in CXCR2 ligands, which induced fibrotic markers of fibroblasts and had the potential to promote the senescence in fibroblasts (108). Furthermore, wounds in diabetic mice treated with the CXCR2 antagonist showed reduction in macrophage senescence and local inflammation and facilitated wound closure, suggesting a novel avenue of targeting the CXCR2 receptor for potential therapeutic developments (108).

Therefore, the senescence of macrophages not only impairs their polarization from a proinflammatory phenotype to one that supports reparative processes, but also affects the wound microenvironment and biological functions of other repair cells through paracrine effects. There is also consensus in the literature that M1 macrophages in diabetic wounds suffer from dysfunctional efferocytosis due to reduced PPAR-γ expression, resulting in increased accumulation of apoptotic cells at the wound site (25, 102, 114). This burden, in turn, augments pro-inflammatory activity and sustains the inflammatory phase (25).

#### Numerous Microenvironmental Biomolecules for Impaired M1 to M2 Polarization

The wound microenvironment is highly complicated, and various factors have been shown to play negative roles in M1/M2 phenotype transition, including metabolic-related outcomes, such as hyperglycemia, advanced glycation end products (AGE), oxidative stress products, and soluble molecules, such as inflammatory factors, neuropeptides, and wound microbes.

The hyperglycemic microenvironment affects the polarization of macrophages in both direct and indirect ways. Huang et al. showed that human monocyte cells (THP-1) were cultivated for 14 days under high or normal glucose conditions. The M1 type marker, CCR7, was significantly upregulated in THP-1 under the high glucose condition as compared to the normal glucose condition, which suggested a biased M1 macrophage (115). Indirectly, hyperglycemia induced generation of reactive oxygen species (ROS) and elevated methylglyoxal (MGO) levels. M2 macrophages are characterized as reductive macrophages, suggesting the redox regulation in macrophages physiology (116). M2 phenotype activation stimulates increased arginase-1 activity and is accompanied by reduced ROS and NO generation. Sustained high levels of oxidative stress obstruct the M1/M2 polarization (117). The mutation of Nox2 or silenced p47 has been shown to inhibit NADPH oxidase to reduce the production of extracellular ROS, favoring the macrophage poise towards the M2 phenotype (117). An elevated MGO usually results in glycation and the increase of AGEs (118). Macrophages express M1 phenotype markers and secrete proinflammatory cytokines after treatment with AGE through activation of the MAPK pathway. Furthermore, AGE was found to induce M1 polarization *via* regulation of PDK4 (119).

As mentioned above, high glucose, AGE, or oxidative stress are known to inhibit the M1/M2 polarization and promote the expression of proinflammatory cytokines in over-activated M1 macrophages. Diabetic subjects have higher serum levels of TNF-a, MCP-1 that is associated with failure to heal in diabetic foot wounds (120). Further, these proinflammatory can form a positive feedback loop to sustain a persistent proinflammatory wound macrophage phenotype. For example, Mirza et al. found that interleukin-1β (IL-1β) and TNF-α as proinflammatory factors are increased in diabetic wound macrophages in both mice and humans (43). BM–derived macrophage exhibited a proinflammatory wound macrophage phenotype when cultured with conditioned medium of chronic wounds, supporting the notion that proinflammatory mediators are involved in the persistent inflammatory phenotype of macrophages in wounds (43). Therefore, a high proinflammatory environment is observed in chronic wounds, and inflammatory factors have the potential to impede M1/M2 polarization of macrophages which, in turn, contribute to sustaining the proinflammatory environment.

The increased accumulation of proinflammatory activity in wounds is derived from adipocytes due to their hypertrophy under diabetic condition (18, 121). Recently, numerous discoveries supported the concept that diabetes and metabolic syndrome are systemic inflammatory diseases (121). This strong association with adipocyte hypertrophy leads to lipotoxicity and excessive production of chemokine and cytokines (122). Moreover, the overexpansion of adipocyte triggers a stress state and eventually results in apoptosis to release inflammatory mediators and attract macrophages to the adipose tissue (123). This causes further infiltration of inflammatory cells, intensifying the secretion of inflammatory mediators, creating a systemic low-grade inflammatory state that may participate in high inflammation states of chronic wounds (124). Consequently, anti-inflammatory treatments may not only improve senescence and insulin resistance but also play an active role in accelerating wound repair.

Neuroregulatory factors are likewise altered in the wounds of diabetics. The expression of neuropeptides was reduced in the skin of diabetic rabbits, and it was accompanied by a chronic proinflammatory state, as indicated by a high M1/M2 macrophage ratio and an elevated proinflammatory cytokine expression, as well as impaired wound healing (125–127). However, the definite molecular mechanism remains unclear.

Chronic wounds are frequently accompanied by the invasion and infection of bacteria due to their unique microenvironment. Multiple species of bacteria have been isolated from chronic wounds, and several also affect the M1 to M2 polarization of macrophages (128). For example, *Pseudomonas aeruginosa* (*P. aeruginosa*) is a frequently detected gram-negative pathogen in diabetic non-biofilm wounds (129, 130). It prolongs the presence of M1 macrophages in chronic wounds in two ways. *P. aeruginosa* produces LPS and binds to the TLR4 receptor complex in macrophages to promote the secretion of inflammatory cytokines (131). Moreover, the type III Secretion System (T3SS) virulence structure is a common trait among all *P. aeruginosa* clinical isolates, which functions as a conduit to directly translocate effector toxins into the target cells and result in inflammation (132). Therefore, *P. aeruginosa* affected wound healing may involve increasing the number of M1 macrophages in wounds accompanied by elevated pro-inflammatory cytokine production.

In summary, the unique and complex wound microenvironment of chronic wounds inhibits the polarization of macrophages from the inflammatory to the repair phenotype. Therefore, the inflammation phase of healing cannot transition to the proliferative phase in chronic wounds, and the proliferation of connective, endothelial, and epithelial tissue cannot be further completed (52). Consequently, targeted changes in the unbalanced microenvironment of chronic wounds may be of great significance to promote healing. Simultaneously, the imbalanced microenvironment changes have multiple-aspects and levels, determining that the multi-target treatment may be more effective than the single-target treatment in local wound treatment.

## Biological Treatment for Restoration of Macrophage Function

Sustained increases in the number of wound macrophages and the dysregulation of their phenotypes, caused both by intrinsic alterations in HSPCs and by a local sophisticated microenvironment, lead to impaired wound healing. Recently, several therapeutic approaches aimed at restoration of macrophage function have garnered significant attention. Methods include systemic treatment *via* oral administration of drugs and local treatment through neutralizing antibodies, MSCs, and biomaterials are discussed.

### Systemic Treatment Strategies

As mentioned in the previous section, the majority of macrophages in wounds are derived mainly from HSPCs. These already predisposed progenitor cells are partially responsible for the dysregulated macrophage polarization and prolonged inflammation in chronic wounds, which suggests that a HSPCs based therapy may improve the function of macrophages from the source.

Docosahexaenoic acid (DHA) is an omega-3 fatty acid, which is prerequisite for cell growth, development, and metabolic functions in mammals. It is also the precursor of several molecules that regulate the resolution of inflammation. The impairment of DHA synthesis affects macrophage plasticity and polarization both *in vitro* and *in vivo* through an Elovl2 (Elovl2−/−) deficient mice model, suggesting a potential role of DHA in regulating the function of macrophage progenitor cells (133). Further, Jia et al. have demonstrated that the accumulation of total macrophages (CD68+) in the wounds of diabetic rats treated with oral DHA remained unchanged, but there is a higher ratio of M2/M1 phenotype compared with diabetic rats, thereby promoting resolution of inflammation in diabetic wounds and accelerating wound healing. Moreover, *in vitro* differentiation experiments confirmed that secretory features of M1 and M2 macrophages differentiated from bone marrow-derived macrophages in the oral DHA group were similar to those in normal rats, but different from those of M1 and M2 macrophages in the diabetic group with more iNOS, TNF-α, IL-1β, and interleukin-6 in M1 macrophages and less Arg-1, interleukin-10, and TGF-β1 in M2 macrophages. The same study implies that oral DHA in diabetic patients is able to correct the impaired plasticity of HSPCs, thereby improving resolution of inflammation, stimulating the transition into the proliferation stage, and thus promoting wound repair (134).

Chronic low-grade systemic inflammation is a common pathophysiological property of aging, obesity and diabetes (82, 135, 136). It is associated with a higher content of pro-inflammatory macrophages (137–139). Prolonged and persistent systemic inflammation can be destructive to various tissues and impair wound healing (140). Consequently, anti-inflammatory strategies focus on the inflammation resolution to stop or dampen the inflammatory response, which may ameliorate the systemic and local inflammation state. Apart from treatment of the primary disease, such as diabetes, improving complications caused by it, such as chronic wounds, requires further study.

### Local Treatment for Improvement of Unbalanced Microenvironment

#### Pro-Inflammatory Microenvironment-Directed Approach

Over-activated M1 macrophages contribute to the hyper-inflammation state of chronic wounds by secreting high levels of pro-inflammatory factors. Neutralizing antibodies aim against these factors that silence M1 macrophages are a promising strategy for enhancing wound healing. Goren et al. found that on the seventh day of post-wounding in diabetic wounds, at the end of the inflammatory period, the administration of anti-TNF-a or anti-F4/80 antibodies promotes re-epithelialization and closure of the wound, whereas the wound in the non-treatment group remains unhealed with scabs. Decreased levels of TNF-a, IL-1β, and CCL2 (MCP-1) proteins were also found in wounds with anti-TNF-a or anti-F4/80. Overall, anti-TNF-a and anti-F4/80 therapies reduced the impact of M1 macrophages, and accelerated the healing of diabetic wounds (141). Furthermore, sustained expression of IL-1β in both of diabetic wounds mice and humans impaired the activity of PPAR-γ, which was closely associated with the switch in macrophage phenotypes. Therefore, blocking the proinflammatory cytokine IL-1β at the local sites or topical administration of PPAR-γ agonists promoted a pro-healing macrophage phenotype and accelerated wound healing (43, 102, 142). Further, Arginase, as a specific phenotypic marker of M2 macrophages, is also widely expressed in other wound cells including keratinocytes, fibroblasts, and endothelial cells. It has been found to play critical roles in inflammatory response and cellular functions through controlling the local arginine concentration and regulating nitric oxide production (143, 144). Decreased arginase level has been found in tissue of chronic non-healing (145). And local inhibition of arginase activity by genetic and pharmacological means significantly impedes wound repair with enhanced inflammatory infiltrate and delayed re-epithelialization (146). Contrastly, Kavalukas et al. demonstrated that depression of arginase through a specific inhibitor 2(S)-amino-6-boronohexanoic acid NH4 (ABH) significantly accelerated wound closure accompanied by increased granulation tissue formation and enhanced re-epithelialization (147). The inconsistency of these results may be related to the model used in the research, the means and degree of inhibiting arginase activity. Therefore, treatment strategies based on arginase needs further research.

Peace or inhibition of overactivated signaling pathways is another alternative approach. The AGE-RAGE signaling pathway is significantly enhanced in diabetic wounds, inhibiting macrophage polarization and M2 phenotypic macrophage function (148). Anti-RAGE antibody-applied wounds increased the number of neutrophils phagocytized by macrophages and promoted the phenotypic switch of macrophages from proinflammatory to pro-healing activities (148).

Oxygen radical scavengers have been reported to provide positive effects in the treatment of chronic wounds (149). For example, topically applied mepenzolate bromide to the wound bed considerably corrected the excessive and prolonged ROS production, and further decreased the level of pro-inflammatory cytokines and increased the level of pro-healing cytokines, results in a promotion of macrophage M2 phenotype polarization, thus accelerating the wound closure rate (150). Antioxidants *α*-tocopherol and N-acetylcystein showed a strong oxidative stress clearance through inhibition of the activity of two antioxidant enzymes, GPx and catalase. Chronicity was reversed in non-healing wounds by treatment with these antioxidants (151). Moreover, natural or synthetic antioxidant compounds, such as sulforaphane (SFN), wogonin (WG), oltipraz (OTZ), and dimethyl fumarate (DMF) are used to evaluate their effect on macrophage polarization. They could elicit phenotypic changes from the M1 gene signature to M2 gene signature *via* indication of the nuclear factor erythroid 2-related factor 2 (Nrf2), a key member of antioxidant regulators (152).

The treatment strategies presented in this review are therefore presented principally to weaken and combat the adverse factors in chronic wounds, balance the inflammatory wound microenvironment, promote the polarization of macrophages, and finally facilitate the wound repair process into the proliferative stage.

#### Macrophage-Directed Gene Therapy Approach

CD163 has been proposed as a specific marker for macrophages with an anti-inflammatory phenotype. It has been shown that using a modified nanoparticle, polyethylenimine (PEI) grafted with a mannose receptor ligand (Man-PEI) to induce CD163 in human primary macrophages lead to changes in the secretion profile and induced anti-inflammatory responses (153). Further, Ferreira et al. likewise used this cell-directed nanotechnology to induce expression of the CD163 gene in THP-1 and human primary macrophages. They found that polarized M2 macrophages have the ability to promote a faster wound healing by interacting with keratinocytes and fibroblasts (154). This precise targeting method can avoids limitations and side effects due to the heterogeneity of chemical-based therapy on different cells and may become an important strategy to reverse chronic wounds.

#### Exogenous Cell Supplement Approach

Chronic wounds are characterized by the polarization barrier of macrophages leading to a higher proportion of M1 phenotypic macrophages and a lower proportion of M2 phenotypic macrophages. A direct addition of exogenous M2 macrophages to the wound may promote wound repair. For example, after activation by hypoosmotic shock, peripheral blood-derived macrophages exhibited anti-inflammatory features and subsequently secreted a large number of repair-related signature genes, such as TGF-β, FGF-8, TNF receptors, VEGF, and GM-CSF (155). These activated macrophages with anti-inflammatory properties may be beneficial for wound repair. Presently, this method lacks sufficient supporting data, and further research is required.

Mesenchymal stem cells (MSCs) are high multi-potentiality residing in different tissues including BM, adipose tissue, the umbilical cord, and skin (156, 157). In addition to their self-renewal and differentiation capacity into several lineages, their secreted products’ impact on a variety of resident and recruited cells are essential for tissue homeostasis and wound repair, and have therefore been explored as cell therapies (10, 156). Their application in acute and chronic wound models has been successful, resulting in resolution of wound inflammation, and enhancement of angiogenesis and acceleration of wound closure (158–161).

Evidence indicates that MSCs exert powerful modulating effects on the immune system, in particular with regard to the immunoregulatory function on macrophages (162). One of the mechanisms of MSC action on macrophages is *via* their recruitment. For example, BM-MSCs conditioned medium significantly accelerates migration of macrophages *in vitro* (163). Subcutaneous injection and topical application of BM-MSCs conditioned media in wounds increased proportions of macrophages and endothelial progenitor cells compared with control group, thus enhancing wound healing (163). Macrophage recruitment by MSCs may be attributed to high levels of secreted chemoattractants CCL3 (MIP-1a), MIP-2, and CCL12 (MCP-5) (10). Another mechanism of MSC action on macrophages is by augmentation macrophages to engulf apoptotic neutrophils, which is attributed to upregulation of the intercellular adhesion molecule-1 (ICAM-1) on macrophages or enhanced release of soluble extracellular superoxide dismutase (SOD3) from MSCs (164–166). Another mechanism of MSC action on macrophage is *via* enhancement of M1-M2 polarization and increasing the frequency of M2 macrophages in wounds. Several *in vitro* studies showed that macrophage facilitated differentiation into M2 phenotypes when co-cultured with MSCs or MSCs derived secretomes (167–170). MSC-educated macrophages exhibited secretory characteristics of M2 phenotype with increased expression of IL-6 and IL-10 and decreased expression of TNF-α and IL-12 (171). Furthermore, MSCs were found to regulate the macrophage phenotype *in vivo* (169). MSCs or MSCs conditioned medium treatment of wounds produced high levels of IL-10 and VEGF but low levels of TNF-α and IL-6 and induced an accumulation of M2 macrophages in diabetic mice (171, 172). A panel of MSC secreted mediators were studied for their immunomodulatory mechanisms. For example, prostaglandin E-2 (PGE-2), a secreted mediators from MSCs, had a direct effect on the macrophages M1-M2 polarization (169, 173). This process is mainly regulated by PGE2 binding to the EP4 receptor on M1 macrophages, and it involves two pathways including CREB and PI3K signaling (169). Furthermore, MSCs released of the tumor necrosis factor-α (TNF-α)-stimulated protein 6 (TSG-6) increased when they were co-cultured with activated macrophages. Topical delivery of MSCs also resulted in TSG-6 release at wound sites, and suppressed TNF-α to play an immunosuppressive role *in vivo* (174). Other possible mechanisms must be further developed and validated.

Currently, MSCs resolve the unrestrained and prolonged inflammation of chronic wounds in terms of numerous aspects, and are ideally suited for the treatment of chronic wounds (156). Further, MSCs have a unique property, namely, the capacity to sense the microenvironment in which they are located, which determines that its immunoregulatory function of MSCs reveals high plasticity (156, 175). Compared with the chemical-based or growth factor-based strategies, this marks an excellent property and allows for refinement of MSC-based cell therapies in the future (176, 177).

#### Immunomodulatory Biomaterials-Based Approach

Biomaterials can provide suitable environments that enhance inherent biological activities and functions in repairing cells through appropriate biochemical cues (e.g., composition and surface chemistry) and biophysical cues (e.g., stiffness and surface topography) (178–180). An increasing amount of evidence shows that they can also influence the flexible nature of macrophages in wounds.

Immunomodulatory biomaterials, particularly the natural biomaterials, have shown regulation of the macrophage fate (181, 182). The decellularized dermal scaffold (DDS) is a skin tissue that removes cellular components and retains the ECM structure, which was shown to play a therapeutic role in wound repair (183). DDS in particular can regulate the transition of macrophages from the M1 pro-inflammatory phenotype to the M2 pro-repairing phenotype, thus promoting macrophage polarization (184, 185). He et al. suggested that amino acids produced by collagen degradation in DDS activated the acid-sensing pathway in macrophages and induced fate transition (184). Further, the inherent components of the ECM, such as hyaluronic acid (HA), or the analogues of the ECM including chitosan, influence the M1-to-M2 phenotype switching (185, 186). Physical and chemical properties of biomaterials have a profound impact on the cellular behavior. For example, a high molecular weight of HA caused macrophages to take on anti-inflammatory features, whereas a low molecular weight of HA resulted in the activation of M1 macrophages (187–189). Biomaterials composed of collagen and highly sulfated HA derivatives promoted switching from the M1 to M2 phenotype of macrophages. Keratin biomaterials have also been used in wound repair studies, and their ability to tune inflammation has been confirmed (190–192). *In vitro* studies have shown that primary macrophages inoculated into high molecular weight extracted keratin and keratin peptide coatings facilitated differentiation into M2c macrophages with anti-inflammatory behavior (190). Chitosan is similar to glycosaminoglycan in ECM, and filmed chitosan can promote higher macrophage production of IL-10 and TGF-β1 anti-inflammatory cytokines as an indication of the M2 phenotype (193). Therefore, immunomodulatory biomaterials with the goal of promoting macrophage polarization present an innovative repair strategy for chronic wounds.

Biological materials not only have the ability of immunological regulation, but also serve as carriers of cells and active molecules to enhance survival rates of transplanted cells and avoid the burst release of active molecules and rapid degradation. Therefore, combining the above-mentioned immunomodulatory biomaterials with active molecules that promote the polarization of macrophages to M2 phenotype may achieve a better pro-healing function than using materials or cells or active molecules alone. An increasing body of evidence points to the benefits of such approaches in inert implants (189, 194). Bioactive molecules or cells have been pursued to enhance wound repair, and they have been successfully administered through the different delivery systems, such as sponge scaffolds (195, 196), polymer (197, 198), hydrogel (199–201), and nanofibers (202, 203), which prove to be effective in preclinical studies.

Notably, increasing evidence reveals the immunomodulatory effects of biomaterials, which may become important approaches of treating chronic wounds, while the mechanism responsible for this response has rarely been explored. In future research, a large number of studies are required to examine the potential mechanism of immunomodulatory materials, and offers guidance for the design of more desirable biological materials to improve tissue repair and regeneration.

## Perspectives

Based on this evidence, focus on the restoration of normal conversion of M1/M2 macrophage phenotypes is beneficial to proper wound healing. Notably, the therapeutic perspective and specific signal targets differ in the existing methods. We consider using existing multiple treatments in combination to achieve synergistic effects between each other, striving for the better therapeutic effects for the treatment of chronic wounds. Arguably, this is insufficient. The development of macrophage-specific pro-healing strategies is only the beginning. Several issues remain to be explored, including the modulation of macrophage activation and resolution of inflammation, the heterogeneity of the macrophage and discovery of novel subpopulations during the repair process, biological functions of individual macrophage lineages and the interaction mode between each subgroup or between each subgroup and other repair cells, and the understanding of the individual cues that can manipulate this heterogeneity. More importantly, specific molecular targets capable of repairing or restoring dysfunctional macrophages must also be intensively investigated. Novel technical approaches may be expected to solve these problems and find possible answers.

Advances in single cell RNA-sequencing (scRNA-Seq) offer an unprecedented approach for its power in monitoring the temporal evolution of heterogeneity and helping to identify novel cell subpopulations and unveil cellular interactions (204, 205). This methodology is increasingly employed in fields such as stem cell biology and oncology, while its applications in wound repair are still limited. Lately, Haensel et al. used scRNA-Seq analysis to identify epidermal hierarchical-lineage and transitional states during normal homeostasis and wound healing (206). Furthermore, Mahmoudi et al. revealed that the distinct subpopulations of fibroblasts with different cytokine expression and signaling in the wounds of old mice compared with young mice (207). Guerrero-Juarez et al. reported that fibroblasts, a major type of repair cells in wounds, grouped into twelve subsets, which demonstrated the high degree of heterogeneity among fibroblasts (208). Moreover, one cluster of fibroblasts might originate from myeloid cells, according to the lineage tracing experiments. These results suggest the possibility and importance of scRNA-Seq technology as a unique tool for the better understanding of chronic wounds and therefore the development of macrophage-specific pro-healing strategies.

**Figure 3:**
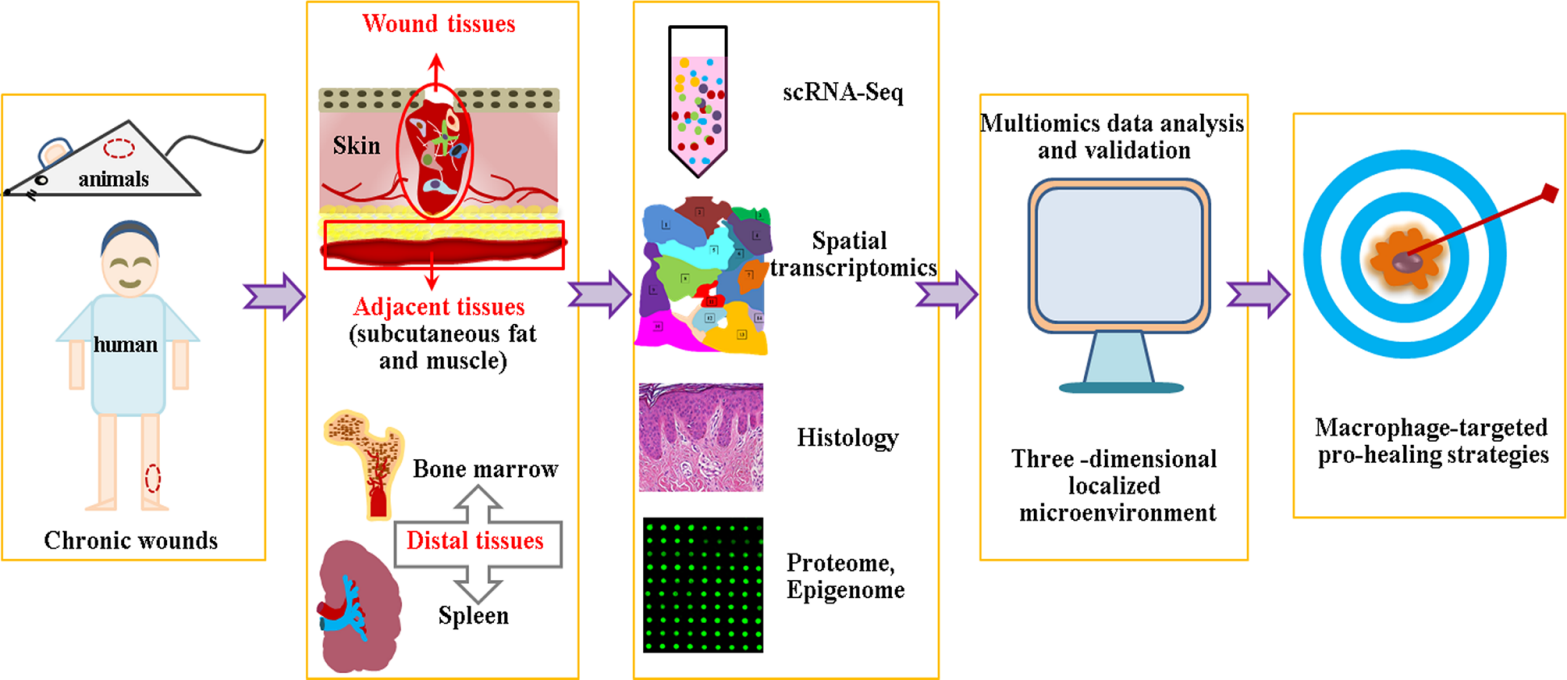
Multiomics data analysis based macrophage specific therapy.

Certain limitations remain in the scRNA-Seq technology, including dropout events caused by the low detection efficiency of non-coding RNA or the failure of amplification of the original RNA transcripts (209–211), which may lead to misinterpretation of data in the downstream analysis. Novel single-cell transcriptome profiling or unique computational method must be developed to address this problem. Another issue is scRNA-Seq deciphering at a transcriptome level, as it is difficult to examine the abundance and post-translation of proteins, or provide spatial information of individual cells. The integrative analysis of multiomics data, such as scRNA-seq, spatial transcriptomics, proteome, histology and epigenome, etc., is to build a three -dimensional localized microenvironment conducive to resolve this question (212). An additional problem is the integration principle, as the occurrence of chronic wounds is affected by both local and systemic factors. Hence, we must not only pay attention to the local pathophysiology of chronic wounds, but also concern ourselves with adjacent tissues, such as subcutaneous adipose tissue or muscle, as adipocytes are important participants in hair follicle regeneration (213, 214). Furthermore, researchers must also focus on the pathophysiology of distal tissues, as aging and diabetes are accompanied by the abnormal output and function of immune and stem cells (215–218) (**Figure 3**).

## Conclusion

Macrophages play essential roles in the persistence of the initial inflammatory process in chronic wounds. Hence, the treatment of chronic wounds lies in immunomodulation. Sustained increase in the number of wound macrophages and impairment of phenotypic switching are caused both by intrinsic alterations of HSPCs and an imbalanced wound microenvironment, which suggests that the treatment of chronic wounds requires consideration of multiple factors. Furthermore, high-throughput methods are a favorable tool to investigate the pathogenesis in detail and discover new targets, although their results require further validation. Moreover, it is a remarkable fact that there are differences in the thickness and number of cells between mouse and human skin, suggesting that skin wound healing in mice may differ from that in humans. Macrophage surface markers have been shown to differ in mice and humans (219). These variations make murine system difficult to translate to human conditions. In future studies, it is necessary to further validate prospective studies in humans to fill the gaps between pre-clinical and clinical studies.

## Author Contributions

ML: Writing-reviewing and editing. QH: Literature investigation and collation. LZ: Literature investigation and collation. YZ: Pictures in the article. XF: Conceptualization. All authors contributed to the article and approved the submitted version.

## Funding

This study was supported in part by the National Nature Science Foundation of China (81971841, 81830064, 81721092, 81941021, 81901973), the National Key Research and Development Plan (2017YFC1104701, 2017YFC1103304, 2018YFC2000400), the CAMS Innovation Fund for Medical Sciences (CIFMS, 2019-I2M-5-059) and the Military Medical Research and Development Projects (AWS17J005, 2019-126). The National S&T Resource Sharing service platform Project of China (YCZYPT[2018]07), and the General Hospital of PLA Medical Big Data R&D Project (MBD2018030).

## Conflict of Interest

The authors declare that the research was conducted in the absence of any commercial or financial relationships that could be construed as a potential conflict of interest.
